# A Convenient Non-harm Cervical Spondylosis Intelligent Identity method based on Machine Learning

**DOI:** 10.1038/s41598-018-32377-3

**Published:** 2018-11-27

**Authors:** Nana Wang, Xi Huang, Yi Rao, Jing Xiao, Jiahui Lu, Nian Wang, Li Cui

**Affiliations:** 1 Institute of Computing Technology(ICT), Chinese Academy of Sciences(CAS), Beijing, China; 2 University of Chinese Academy of Sciences, Beijing, China; 3 Xiyuan Hospital, China Academy of Chinese Medical Sciences(CACMS), Beijing, China

## Abstract

Cervical spondylosis (CS), a most common orthopedic diseases, is mainly identified by the doctor’s judgment from the clinical symptoms and cervical change provided by expensive instruments in hospital. Owing to the development of the surface electromyography (sEMG) technique and artificial intelligence, we proposed a convenient non-harm CS intelligent identify method EasiCNCSII, including the sEMG data acquisition and the CS identification. Faced with the limit testable muscles, the data acquisition method are proposed to conveniently and effectively collect data based on the tendons theory and CS etiology. Faced with high-dimension and the weak availability of the data, the 3-tier model EasiAI is developed to intelligently identify CS. The common features and new features are extracted from raw sEMG data in first tier. The EasiRF is proposed in second tier to further reduce the data dimension, improving the performance. A classification model based on gradient boosted regression tree is developed in third tier to identify CS. Compared with 4 common machine learning classification models, the EasiCNCSII achieves best performance of 91.02% in mean accuracy, 97.14% in mean sensitivity, 81.43% in mean specificity, 0.95 in mean AUC.

## Introduction

Cervical spondylosis (CS) is a degenerative disorder, an common disease, which affects up to two-thirds of the population in their lifetime^1–4^. The CS seriously affect people’s physical and mental health and quality of life and increase the burden on individuals and society. What’s more, it leads to the loss of human-related functions and is accompanied by depression, anxiety and other psychological damage. The main complaint of CS is neck pain which is reported by approximate 30–50% from the patients^5^. Meanwhile, the global point prevalence of neck pain was 4.9% and the neck pain ranked 4th highest in terms of disability as measured by YLDs, and 21st in terms of overall burden in the Global Burden of Disease 2010 Study^6^. The early detection of the CS is critical for burden lighten. As the earlier the disease is discovered, the easier it is to treat, the higher the cure rate is, and the less the patient spend. The CS is a chronic ‘wear and tear’ degenerative process of the cervical spine that initially is the vertebral bodies and intervertebral disks degeneration in the neck, and can develop into disk ruptures and herniation, osteophyte, compression of the spinal cord, or cervical spondylotic myelopathy (the most important complication of degenerative disease of the cervical spine)^7–9^. As cervical degeneration worsens, clinical manifestations become more obvious, and the difficulty and cost of treatment increases. In detail, for the patients with the vertebral bodies and intervertebral disks degeneration in the neck, non-operative treatment continues to play an important role in treatment^2^. For the patients with cervical spondylotic myelopathy, surgical treatment have been conventional means but may lead to significant problems including adjacent level^2,10^.

The pathogenesis and clinical symptoms of the CS are complex. For the pathogenesis, it is supported by the modern medical theory that the chronic degeneration of the cervical spine lead to the CS. The degeneration cause the changes in the morphological or structural of the spine, such as loss of disc height, disk ruptures and herniation, narrowing of the foramina, osteophyte. Since the morphological structure of the spine can be records by imaging instruments^11–16^, the change above can be directly observed from the imaging information. For example, the oblique radiograph of the cervical spine in a patient with CS shows the loss of disc height, anterior osteophytosis, and narrowing of the foramina^17^. For the clinical symptoms, the clinical symptoms accompanying the occurrence and development of CS are complex and varied^17,18^. In the early stage of CS, the suffers are usually harassed by the neck pain, cervical stiffness and other discomfort in the neck, shoulder blades, and upper limbs. As the disease worsens, the range of neck movement is limited, and the pain is aggravated by the movements. Due to the complex above, the identification of CS is a sophisticated and complicated work. The classification of CS, the clinical symptoms and imaging characteristics are presented in the CS diagnostic criteria. Combined with the professional knowledge as well as clinical experience, the doctor collects relevant symptom information in the form of question and answer, analyze the symptoms and imaging information, and gives a diagnosis result according to the diagnostic criteria. However, when diagnosed by clinical symptoms, the accuracy of the diagnosis result is hindered by the asymptomatic, slight early symptoms that are easily overlooked, individual differences, and unrelated causes (such as the neurological disease). When diagnosed by observing the morphological or structural changes of the spine, the cost of the diagnosis is increased by the expensive medical instrument. When diagnosed by the clinical approach, the timeliness and cost of the diagnosis is affected by the cumbersome diagnostic process, which involves medical instruments resource and need the intervention of doctors or experts. What’s more, the concern on the healthy will be aggravated by frequent inspection of medical instruments, for example the radiation.

It is also agreed by the tendons injury theory of traditional chinese medicine that the pathological changes of the neck muscle system (the tendons of neck) is the causative factor of CS. In detail, the pathological changes of the cervical muscle system leads to the decline in its own mechanical properties, the destruction in the exogenous stability of the cervical vertebrae, aggravate the degeneration of the cervical spine, and finally form the CS^19^. The pathological changes of the neck muscle system and cervical spine cause chronic dysfunction as well as pain^20^, leading to anomalous pattern of muscle activity^21–27^. And when the muscles are activated in the activity, the Motor Unit Action Potential Trains (MUAPTs) are generated by motor units, superimposed on the surface of the skin and form a non-stationary week signal which can be acquired by the sEMG device and generate electromyography. Therefore the pathological changes can be captured by the sEMG device. Meanwhile, there are many researches on using myoelectricity to study the muscle activity and functional status^21–27^, which demonstrates that there are differences in sEMG signals between population with cervical musculoskeletal disorders or neck pain and the healthy. What’s more, the tender point refers to the area where pain can occur when pressed, the distribution of which is closely related to biomechanics^28^. It is the signs of neck muscle pathological changes^19,29^. The tender points of patients with CS mostly located on the cervical paravertebral muscle, trapezius and sternocleidomastoid muscle^30^. Thus, it provides a chance that we can explore the relationship between sEMG signals on the muscles of the cervical paravertebral muscle, trapezius and sternocleidomastoid muscle and CS to identify CS. Benefit from the development of sensors technology, sEMG device become more portal and more cheaper, promoting the sEMG technology to become a competitive choice for the convenient CS identification. The sEMG have attracted a lot of attention in muscle function assessment^31,32^, muscle activity assessment^23,24,26^, rehabilitation effect tracking^33^ and rehabilitation guidance^34^.

Meanwhile, the statistical analysis also opens a window onto wellness^35^. The traditional statistical analysis methods, has been widely used in clinical data analysis, for instance linear or logistic regression^36–38^. As the increasing data become larger and complicated, modern statistical methods have been used to deal with the complicated data^39^. The machine learning, a powerful analysis methods, has made great progress in medical^40–45^. With the convenient of sEMG technology and the development of artificial intelligence, the convenient, no-harm, intelligent CS identification method can be considered. The data acquired by portal sEMG device present a huge challenge to the identification of CS. The high-dimensional sEMG data can cause dimensional disaster which decrease computational efficiency, increase memory storage requirements, and cause overfit. Faced with the high-dimensional data, feature extraction and feature selection, which are effective means of data preprocessing, have the advantages of improving model performance, increasing computational efficiency, decreasing memory storage requirements, giving model better readability and interpretability, and building better generalization model^46^. Besides, the data shows weak availability of faulty, redundant, insufficient, sparse distribution since the data acquisition is susceptible to external factors in non-lab environments by portal sEMG device. So a powerful machine learning model should be considered. Ensemble learning containing a number of weak learners can not only learn linear and complex nonlinear function but also boost weak learners which are slightly better than random guess to strong learners which can make very accurate predictions^47^. The gradient boosted regression tree (GBRT), one of powerful ensemble learning, has been successfully used in classification task^48–51^. And it is a competitive choice for the classification task on limited weakly available data.

**Figure 1:**
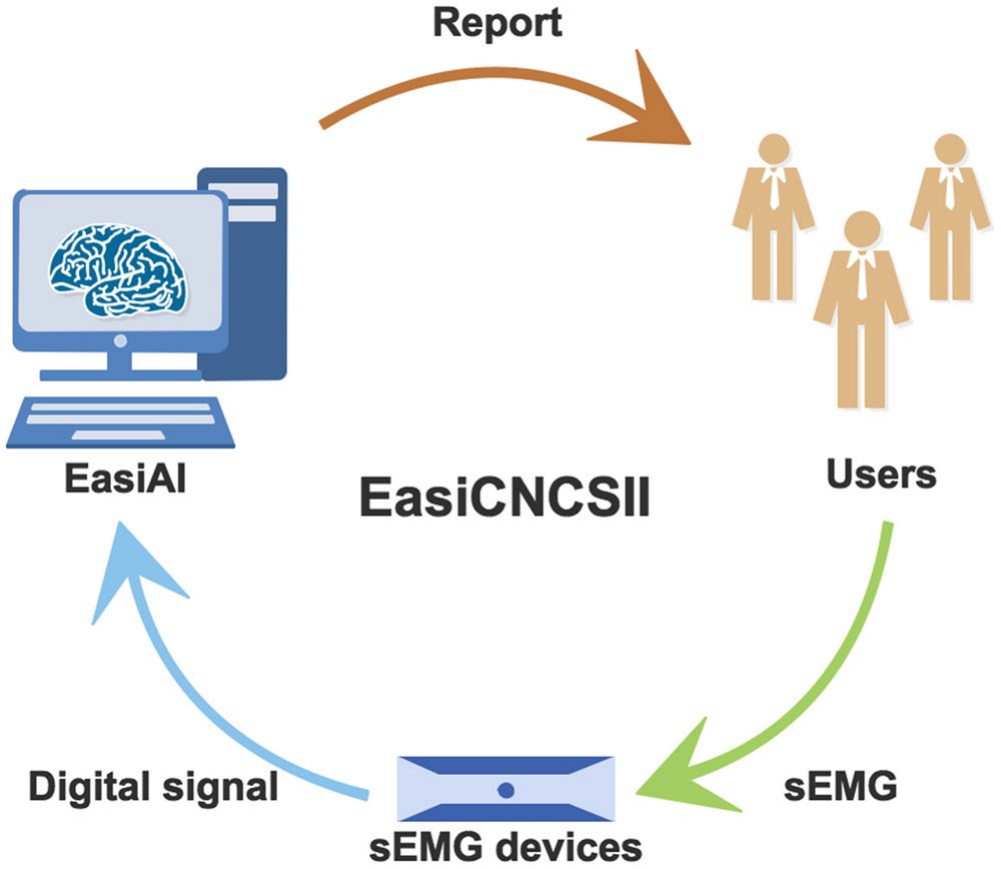
The CS identify based on sEMG and machine learning. Firstly, users perform a set of movements according to instructions. Secondly, the portable sEMG device acquire sEMG signals of the user and send it to the smart terminal. Finally, the EasiAI, an intelligent CS identification model, predict the state of user using the sEMG signals and return the report which shows whether the user suffer from the CS.

In this work, we proposed a new convenient, non-harm and intelligent method EasiCNCSII to identify CS based on sEMG and machine learning as the Fig. 1 shown. The method mainly consist of data acquisition and CS identification. For data acquisition, we proposed a convenient, time-saving data acquisition method, which involves 6 muscles and 7 movements (see Supplementary for the selection of muscles and movements). The user only need to spend less than 20 minutes independently completing a set of simple movements according to instruction, after connecting the portal sEMG device to the laptop and user’s neck muscles. The relevant data is uploaded to the intelligent processing terminal while being collected by the sEMG device. For CS identification, the EasiAI model based on 3-tier architecture, was developed to identify CS. The EasiAI consists of feature extraction, feature selection and classification algorithm. For feature extraction, we extract 11 types of features from raw sEMG signal, of which 6 types are extracted in the common high dimensional time series feature extraction methods, such as time-domain method, of which 5 types are built inspired by the relevant knowledge. Most of the features are proved to be significantly associated with the CS by Pearson (*p* ≤ 0.05). For feature selection, EasiRF, a feature selection method, was developed to select the most relevant features and improve the performance of the CS identification. The easyRF is validated effective compared with traditional feature selection algorithms. For classification algorithm, a classification algorithm based on GBRT is developed to identify CS. The EasiAI achieve the best performance with 91.02% in accuracy, 97.14% in sensitivity, and 81.43% in specificity compared with 4 kinds of machine learning classification model. The EasiCNCSII is validated effective.

## Methods

### Participates

The 179 volunteers participated in the study from March 15, 2017 to December 31, 2017 in China, of which 109 were CS suffers and 70 were the heathy. The former have received a clinical diagnosis of the CS, which are in accordance with 2012 ICD-9-CM Diagnosis Code 721 (721.0 Cervical spondylosis without myelopathy, 721.1 Cervical spondylosis with myelopathy) and the criteria of diagnosis and treatment for CS issued by China Rehabilitation Medicine association. The latter free from the CS are diagnosed by the rich experienced clinicians. The exclusion criteria includes cervical vertebral trauma, cervical vertebral surgery, congenital spinal deformity, syringomyelia, amyotrophic lateral sclerosis, spinal cord tumor, spinal cord injury, adhesive arachnoiditis, the fascitis, cervical injury, tumor or infection, pregnant, breast-feeding, menstruation. All data collection protocols were approved by Institute of Computing Technology, Chinese Academy of Sciences. Written informed consents were obtained from all volunteers. The acquisition method was carried out in accordance with the approved guidelines. See Supplementary for the instruction of data acquisition.

### The EasiCNCSII

**Figure 2:**
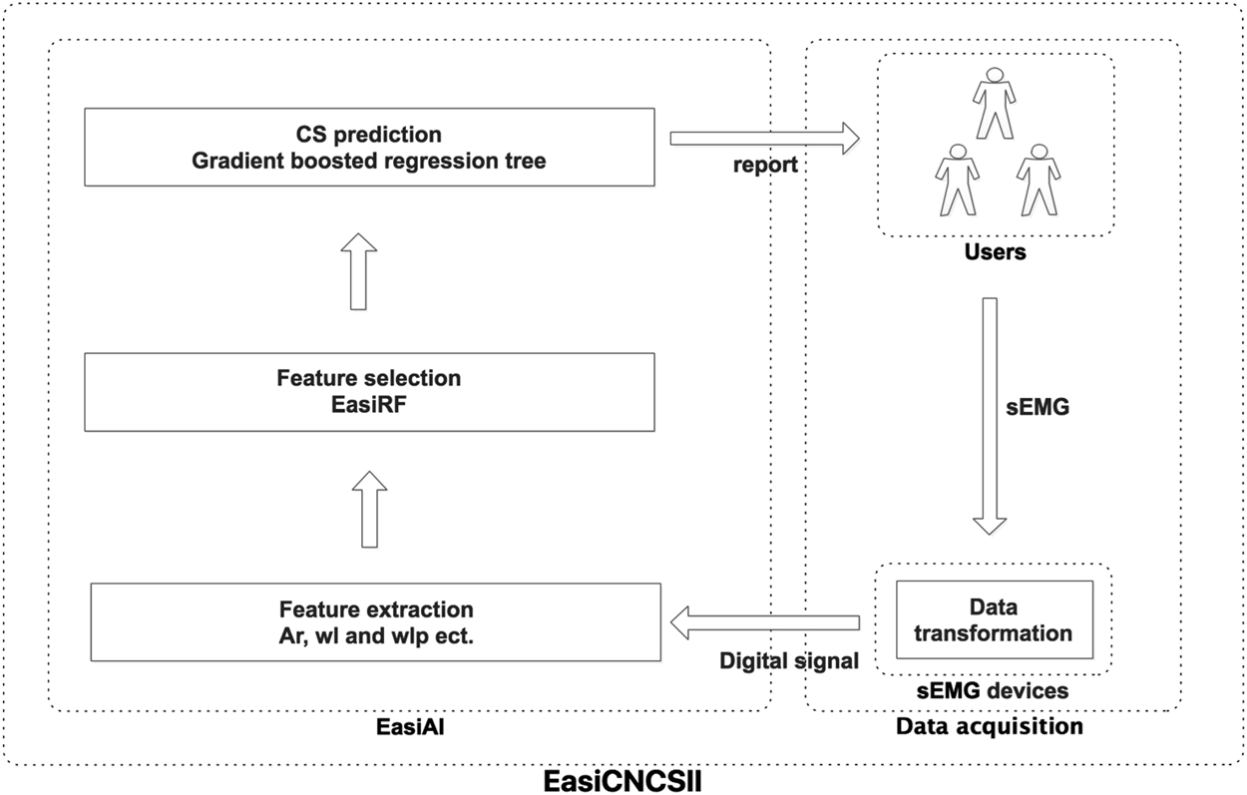
The EasiCNCSII method. The left side of the figure is the CS identify model EasiAI consisting of feature extraction, feature selection, classification algorithm. The right side of the figure is data acquisition. The data collected by sEMG is automatically transmitted to the intelligent terminal equipped with the EasiAI. The report generated by the EasiAI is sent to the users.

The EasiCNCSII is designed as shown in Fig. 2, which includes two parts: the data acquisition and the EasiAI model. For data acquisition, the users, after connected sEMG device with the 6 muscles, complete 7 movements according to the simple instructions. The analog signals from the users are converted into the digital signals, which are high dimensional time series data, and sent to the EasiAI by the sEMG device. The EasiAI is a 3-tiers data processing model: feature extraction, feature selection and classification algorithm. Feature extraction algorithm extracts features from the a user’s digital signals in the methods of time-domain, frequency-domain, time-frequency-domain, etc. Feature selection algorithm EasiRF based on the Random Forest (RF) is developed to selected the most relevant features. The classification algorithm based on the gradient boosted regression tree is developed to identify CS, achieving the good performance on limited data set with relative small computing overhead. Using the input data after feature selection, the report, which show whether the user suffers from the CS, is generated and returned to the user. The lightweight algorithms can be integrated into the user end and quickly send report to users without concerns on privacy.

### Feature extraction

The 5 common methods: time-domain, frequency-domain, time-frequency, parametric model and nonlinear feature analysis are used to extracted features from the sEMG signal which are high-dimensional time series data. Besides, the method of using the disease-related knowledge to construct features are also considered. With the 5 common methods above, we extracted 63 features from *S*_*i*,*j*_, of which 11 are extracted in methods of time-domain^52,53^, of which 14 are computed in methods of frequency-domain^52,53^, of which 23 are computed in methods of time-frequency based on wavelet transform and wavelet packet transform^54,55^, 14 of which are extracted in AR parametric model^56^, of which 1 nonlinear entropy feature^52,53,57^ are extracted. Among the 63 features above, the 5 features including the root mean square (RMS), median frequency (MF), mean power frequency (MPF), the average electromyogram (AEMG), and the integrated electromyogram (IEMG) are common features in clinical research. Besides, considering the relative knowledge of the CS, the 45 features are extracted from *S*_*i*_, of which 2 called as cervical flexion-relaxation ratio (FRR)^58,59^ is commonly used in clinical research and can only be extracted from the *S*_1_, and of which 43 are new features. The 63 features from *S*_*i*,*j*_ and 45 features from *Si* are divided into 11 types: TF, FF, WL, WLP, AR, EY, FRR, DU, ACI, UN, SYM, facilitating statistical analysis. Details on the calculation are shown in the feature extraction part of the Supplementary and Supplementary Tables S1 and S2.

### The EasiRF algorithm

The EasiRF based on the RF is a stochastic model since the samples are randomly selected to generate the tree and the features are randomly selected to be splitting rule. Firstly, we generate 7 different data sets and iteratively use RF to train on each data set. Secondly, set the tree number of RF model different, select the top 25 most important features and merge the features of each iteration until the feature number of the merged set is not growing in each iteration. Finally, the final feature set is generated by merging 7 feature sets from 7 data sets above. As shown in Algorithm 1, the feature selection algorithm EasiRF based on RF is developed to get the most relative features.

**Algorithm 1:**
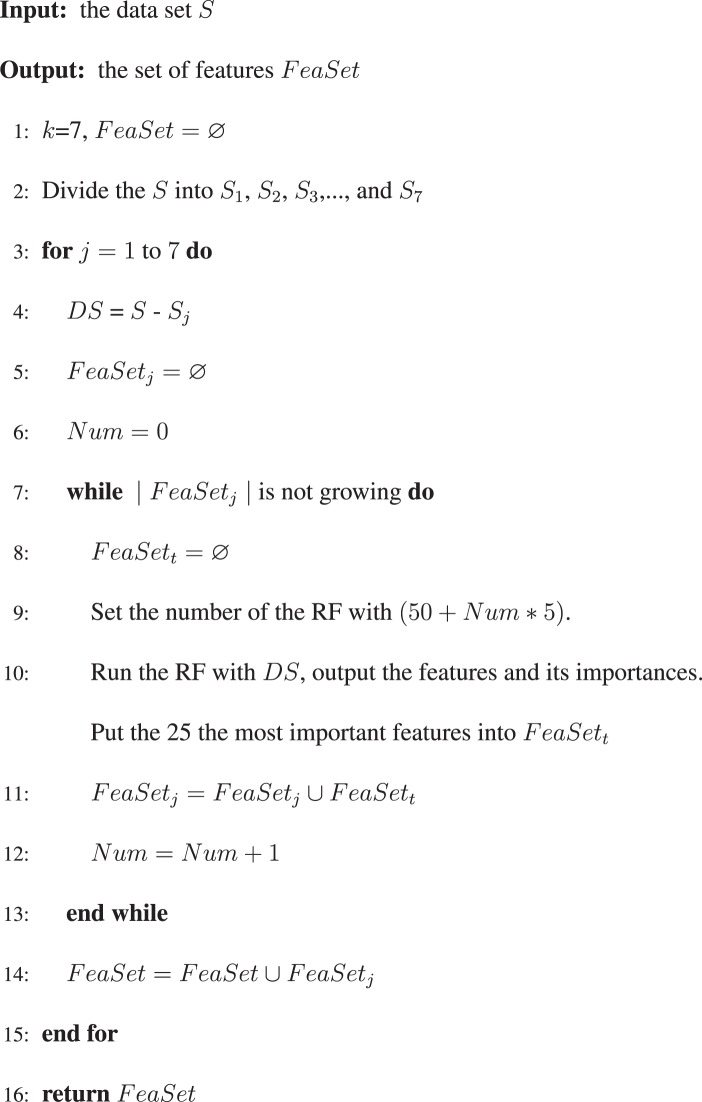
The EasiRF algorithm.

### The classification algorithm

A classification algorithm based on Gradient boosted regression tree (GBRT) is developed to identify CS. The GBRT is also known as gradient boosting machine (GBM) or gradient tree boosting^60^. It is a tree ensemble model and its base classifier is a decision tree that can be used both for regression and for classification. The GBRT model is trained iteratively. For each iteration, the sample instance to minimize the objective is added. In detail, if a given dataset *D* (*D* = (*x*_*i*_, *y*_*i*_), 1 ≤ *i* ≤ *n*, *x*_*i*_ ∈ *R*^*m*^, *y*_*i*_ ∈ *R*) consist of n samples with m features, and ${{\hat{y}}_{i}}^{(t)}$ is the prediction of the i-th instance at the t-th iteration, the GBRT can be trained after t iteration, for each of which we add *f*_*t*_ with the minimum objective function *L*^(*t*)^ value in Formula 1.

1 $${L}^{(t)}=\sum _{i=1}^{n}l({y}_{i},{{\hat{y}}_{i}}^{(t-1)})+{f}_{t}({x}_{i})+g({f}_{t})$$

Here, the *L*^(*t*)^ is the objective function in t-th iteration.

### The metric of model performance

The accuracy, sensitivity, specificity, FNR, FPR and AUC (the area under the sensitivity and specificity curve) are used to evaluate the performance of algorithms in our work in Formula 2–6. The large the accuracy, sensitivity and specificity are, the smaller the FNR and FPR are, the better performance of the model is, the more efficient the feature selection algorithm is. Besides, the larger AUC value indicates higher classification accuracy across a range of threshold choices^40,61^, so the AUC is also used to illustrate the performance of the classifier.

2 $${\rm{accuracy}}=\frac{{\rm{true}}\,{\rm{positive}}+{\rm{true}}\,{\rm{negative}}}{{\rm{positive}}+{\rm{negative}}}$$

3 $${\rm{sensitivity}}=\frac{{\rm{true}}\,{\rm{positive}}}{{\rm{positive}}}$$

4 $${\rm{specificity}}=\frac{{\rm{true}}\,{\rm{negative}}}{{\rm{negative}}}$$

5 $${\rm{FNR}}=\frac{{\rm{false}}\,{\rm{negative}}}{{\rm{positive}}}$$

6 $${\rm{FPR}}=\frac{{\rm{false}}\,{\rm{positive}}}{{\rm{negative}}}$$

The ‘true positive’ is the number of correctly predicted patient with CS, ‘positive’ is the number of patient with CS shown, ‘true negative’ is the number of correctly predicted healthy people free of CS, the ‘negative’ is the number of healthy free of CS shown, the FNR is false negative rate, the FPR is false positive rate, ‘false positive’ is the number of predicted patient with CS which are actually the healthy, and ‘false negative’ is the number of predicted healthy people which are actually the patients with CS. The higher the accuracy, sensitivity and specificity are, the better the performance of model is. The lower the false positive rate and false negative rate are, the better the performance of model is.

### Handling of missing data

Some sample data were missing for some variables. We filled in missing data with the mean value. In detail, the missing data from CS suffers are filled with the mean values of all the samples from the CS suffers. The missing data from health are filled with the mean values of all the samples from the healthy.

### Experimental set-up

The python (version 2.7.13) and matlab (version 2016r) were used to implement features extraction from sEMG data. The python (version 3.6.0) and xgboost (version 0.6) were used to implement the EasiAI model. The RF, NB, LR and SVM model are implemented by the scikit-learn (0.19.1) and the python (version 3.6.0). The laptop and sEMG device which used to collect the sEMG signal are provided by Wireless Sensor Network Lab (this research does not develop the hardware). The EasiAI is deployed in the laptop with the CPU of i7, the memory of 8GB and operating system of 64-bit.

### Code availability

After we reorganize the codes of the EasiAI, the source codes will be available at github. Currently, it is available from wangnana@ict.ac.cn on reasonable request.

### Ethical approval

The study was approved by Institute of Computing Technology, Chinese Academy of Sciences. All volunteers signed informed consents. The acquisition method was carried out in accordance with the approved guidelines.

## Result

### Data preparation

The dataset are made up of 537 samples from 179 volunteers from March 15, 2017 to December 31, 2017 in China, including 109 CS suffers and 70 heathy population. Each volunteer’s sEMG signal was acquired repeatedly 3 times and form 3 samples as shown in Supplementary Fig. S1 (see Supplementary for the instruction of data acquisition). We split the dataset into training samples (training set), validation samples (validation set), and test samples (test set) according to 16: 4: 5. Meanwhile, it is ensured that samples belonging to the same volunteer are only distributed in one of the three sets above. Each sample consists of 7 multiple high-dimensional time series data from a sample, each of which is generated from the movement *A*_*i*_ (the *A*_*i*_ includes bow (*A*_1_), head backwards (*A*_2_), left flexion (*A*_3_), right flexion (*A*_4_), left rotation (*A*_5_), right rotation (*A*_6_), and hands up (*A*_7_)) and represented as *S*_*i*_. The *S*_*i*_ is expressed as Equation 7. The *S*_*i,j*_ denotes the sEMG signals collected from the muscles *M*_*j*_ (the *M*_*j*_ includes left sternocleidomastoid (*M*_1_), left upper trapezius (*M*_2_), left cervical erector spinae (*M*_3_), right cervical erector spinae (*M*_4_), right upper trapezius (*M*_5_), and right sternocleidomastoid (*M*_6_)) activated by movement *A*_*i*_, expressed as Equation 8.

7 $${S}_{i}={[\begin{array}{cccc}{S}_{i\mathrm{,1}}, & {S}_{i\mathrm{,2}}, & \mathrm{...} & {S}_{i,j}\end{array}]}^{{\rm{T}}},\,\mathrm{(1}\le i\le \mathrm{7,}\,1\le j\le \mathrm{6)}$$

8 $${S}_{i,j}=[\begin{array}{cccc}{p}_{1}, & {p}_{2}, & \mathrm{...} & {p}_{n}\end{array}]$$

Here, the *S*_*i,j*_ denotes the electrical signal data produced by the muscle *M*_*j*_ throughout the course of the movement *A*_*i*_ and consists of the *p*_*n*_ which is the value of the float type in python 2.7.

### Feature extraction

For each sample, the 423 (45 + 6 × 63) features are extracted from *A*_1_ and 421 (45 + 6 × 63) features (the 421 features does’t include FRR features) are respectively extracted from the other 6 movements. Thus, 2949 (423 + 6 × 421) features are extracted from the raw sEMG signal generated by all the movements above which include *A*_1_, *A*_2_, *A*_3_, *A*_4_, *A*_5_, *A*_6_, *A*_7_. The 2949 features’s distribution are shown in Supplementary Fig. S3. The Pearson correlation indicated that 1789 features are significantly associated with the CS (*p* ≤ 0.05). (The significant features’s distribution are also shown in Supplementary Fig. S3).

### Feature Selection

We developed a feature selection algorithm EasiRF based on RF to select the most relevant features and improve the performance of the CS identification. The EasiRF divide the 537 samples into 7 different data sets, each of which is represented as *D*_*i*_ (0 ≤ *i* ≤ 7). The RF (The number of trees is set to different value in each iteration.) is iteratively used to select the top 25 most important features from *D*_*i*_. And put the 25 selected features of each iteration into the feature set *S*_*i*_ until the feature number of the *S*_*i*_ is not growing. As shown in Supplementary Fig. S4, the selected feature number tends to be stable on each data set *D*_*i*_ when the iteration number reaches 40. The final feature set including 282 features are generated after merging all the *S*_*i*_ above (the feature type distribution of 282 features are shown in Supplementary Fig. S5).

In order to validate the effectiveness of the EasiRF, the Fisher Score (FS)^62^, Conditional Infomax Feature Extraction (CIFE)^63^, Multi-Cluster Feature Selection (MCFS)^64^, f-score are respectively selected from 4 kinds of traditional feature selection algorithms^46^ as well as EasiRF. With the metrics of accuracy, sensitivity, specificity, FNR and FPR, we compare the performance of the GBRT on test set with the feature selection algorithms above, using five-folds cross-validation. As shown in Table 1, the mean accuracy of the model is 86.54% without feature selection. In spite of the slight drop in mean accuracy with the MCFS and CIFE, the mean accuracy of other algorithms is over 87.10%. What’s more, the mean accuracy with the EasiRF is the highest with the value of 91.02%. The mean sensitivity of the model is 92.51% without feature selection. All the mean sensitivity of the model are over 92.51% with feature selection algorithms. And the mean sensitivity with the EasiRF is the highest with the value of 97.14%. The mean specificity is 77.14% without feature selection. However, the mean specificities with feature selection algorithms are less than 77.14%, except the EasiRF with the highest value of 81.43%. The mean FNR of the model is 7.49% without feature selection. All the mean FNR with the feature selection algorithms are less than 7.49%. The mean RNR with the EasiRF is the smallest with the value of 2.86%. The mean FPR of the model is 22.86% without feature selection. And the mean FPR with feature selection algorithms are larger than 22.86%. The FPR with the EasiRF are the smallest with 18.57%. Compared with the feature selection algorithms above, the EasiRF perform best and are validated most effective. The comparison of more feature selection algorithms are shown in Supplementary Table S3.

**Table 1:** The comparison of performance with different feature selection algorithms.

	Accuracy	Sensitivity	Specificity	FNR	FPR
Non	86.54%	92.51%	77.14%	7.49%	22.86%
FS	87.10%	95.28%	74.29%	4.72%	25.71%
CIFE	86.00%	92.60%	75.71%	7.40%	24.29%
MCFS	83.16%	92.60%	68.57%	7.45%	31.43%
F-score	87.10%	95.28%	74.29%	4.72%	25.71%
EasiRF	91.02%	97.14%	81.43%	2.86%	18.57%

### The train and test of the EasiAI

We train the EasiAI on training set and validate on validation set. The performance of the classifier is mainly affected by model parameters, especially the number of weak classifiers. Thus we first assessed that the number of trees (weak classifiers) included in the EasiAI were enough to obtain the highest AUC on validation set. As shown in Supplementary Fig. S6, the higher the number of trees is, the higher the AUC on the validation set is. Furthermore, the AUC on the validation set achieve highest when the tree number is 535. The other parameters of the EasiAI model are shown in Supplementary Table S4. The final model is generated by training EasiAI using the parameters in Supplementary Table S4 on the dataset consisting of the training set and validation set.

**Figure 3:**
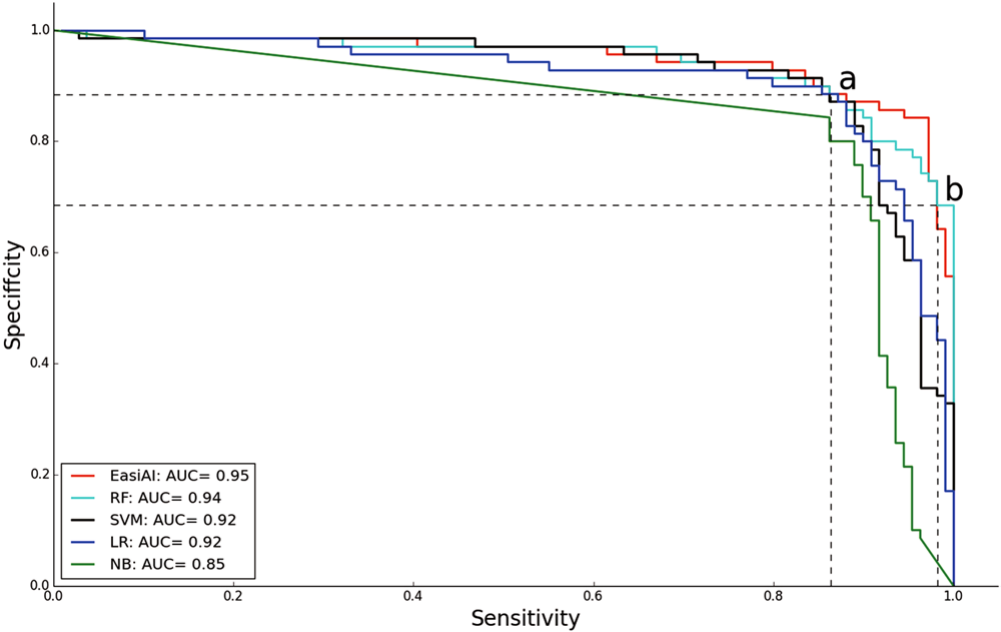
The performances of the EasiAI, RF, SVM, LR, and NB. The x-axis is the sensitivity. The y-axis is the specificity. Different color denotes different machine learning model. The curves of different colors consist of sensitivity and specificity points of different models. The closer the sensitivity and specificity points are to the upper right corner, the large the sensitivity and specificity are, the better the model performs. All the sensitivity and specificity points of the NB are the farthest from the upper right corner and fall below the curves of other models. The two curves of the LR and the SVM are intertwined whose most of sensitivity and specificity points fall below the curve of the RF. The two curves of RF and EasiAI are intertwined and most of the RF’s sensitivity and specificity points fall below the ones of the EasiAI, especially between point a and b. The sensitivity and specificity points of the EasiAI are closest to the upper right corner, and have the highest AUC value 0.95.

To validate the performance of the final EasiAI, we tested the model on test set, using five-fold cross-validation. The metrics are accuracy, sensitivity, specificity, FNR, FPR as well as AUC. The large accuracy, sensitivity, specificity and AUC are, the smaller false negative rate and false positive rate are, the better the model classification performance is. The AUC, the mean accuracy, the mean sensitivity, the mean specificity respectively are 0.95, 91.02%, 97.14% and 81.43% as shown in Fig. 3 and Table 2. The mean FNR, the rate of missed diagnosis, is 2.86%. The mean FPR, the misdiagnosis rates, is 18.57%. Overall, the accuracy of model is higher than 90%, the missed diagnosis rate of our model is less than 3%, and is validated effective.

**Table 2:** The performance of machine learning on test set.

	Accuracy	Sensitivity	Specificity	FNR	FPR
EasiAI	91.02%	97.14%	81.43%	2.86%	18.57%
RF	87.65%	93.42%	78.57%	6.60%	21.43%
SVM	86.00%	89.78%	80.00%	10.22%	20.00%
LR	86.57%	88.92%	82.86%	11.08%	17.14%
NB	82.10%	89.83%	70.00%	10.17%	30.00%

### Comparing with support vector machines (SVM), Logistic regression (LR), NativeBayes (NB), Random Forest (RF)

The identification of CS is a task of classification. Faced with limited data, we chose the following four common models: Support vector machines (SVM), Logistic regression (LR), Native Bayes (NB), and Random Forest (RF). We validate the effectiveness of the EasiAI by comparing the EasiAI with the SVM, LR, NB and RF, on test set (the test set only includes the 282 features which are selected from the 2949 features by the EasiRF) in the same classification task, using five-fold cross-validation. In each fold, we use the gird search approach to get the important parameter value, with which the model perform optimal. The details on the parameters of the EasiAI, SVM, LR, NB and RF are shown in the Supplementary Tables S4–S8. As shown in Table 2, the highest mean accuracy is 91.02% achieved by the EasiAI, and the lowest mean accuracy is 82.10%. The highest mean sensitivity is 97.14% achieved by the EasiAI, and the lowest mean sensitivity is 88.92%. The highest mean specificity is 82.86% and the lowest mean specificity is 70.00%. The mean specificity of the EasiAI is 81.43%, just smaller than the highest. The highest mean FNR is 11.08% and the smallest value is 2.86% achieved by the EasiAI. The highest mean FPR is 30.00% and the lowest value is 17.14%. The mean FPR of the EasiAI is 18.57%, just larger than the smallest one. Besides, the curves of sensitivities and specificities of models above are shown in Fig. 3. Most of the sensitivity and specificity point falls below the red curve of the EasiAI, especially between point a and b. The EasiAI achieve the best performance compared with RF, SVM, LR and NB with the metrics above.

### Analysis of learned knowledge about CS

The EasiAI achieved high accuracy in identifying CS, so we believe that the features in final model play an important role in the classification. And the informative knowledge about the CS can be learned from the feature distribution in muscles and movements.

The number of features and feature importance are two important attributes. The feature number is the number of features extracted from muscles or movements. The more the features that are distributed on the muscle/movement are, the stronger the muscle/movement have the ability to identify CS, the more the muscle/movement have differences between the healthy free from the CS and the CS suffer, the more the muscle/movement is related to CS. The feature importance is the contribution to the performance of task above in the training model. The more important the features from the muscle/movement are, the stronger the muscle/movement have the ability to identify CS, the more the muscle/movement have differences between the healthy free from the CS and the CS suffer, the more the muscle/movement is related to CS.

**Figure 4:**
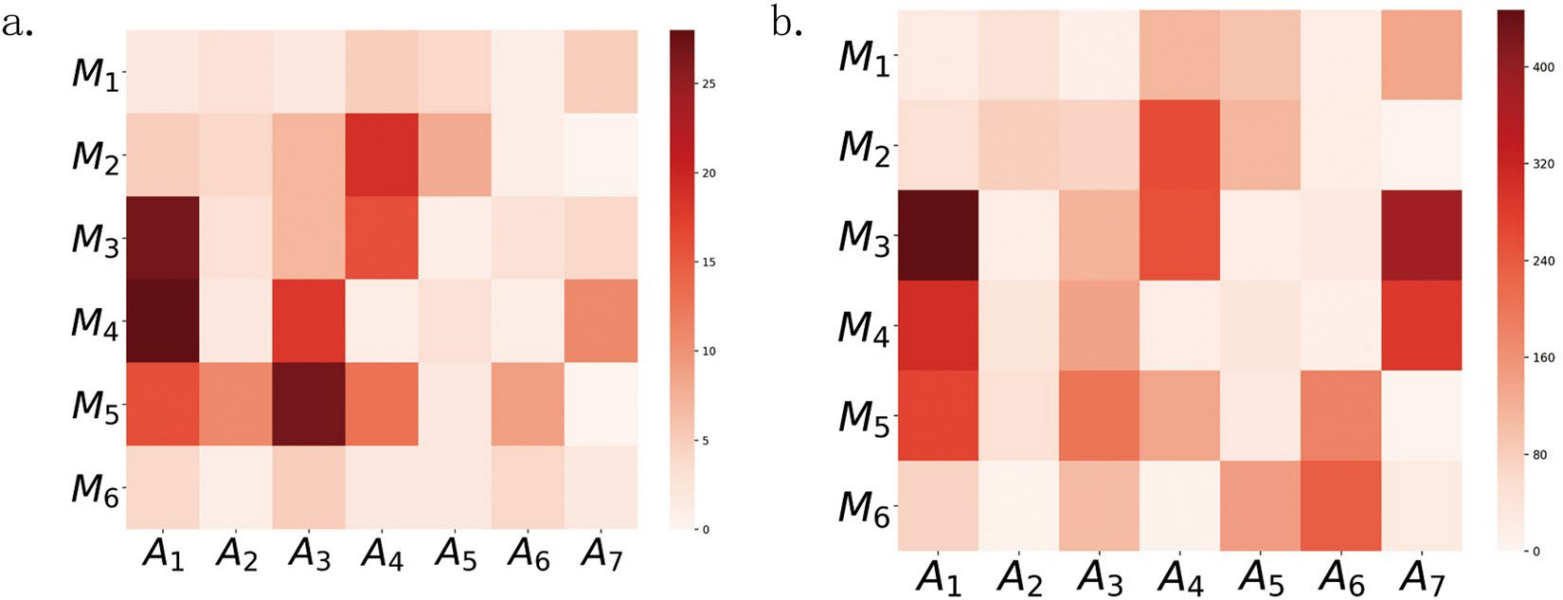
The heat map. The *M*_1_, *M*_2_, *M*_3_, *M*_4_, *M*_5_, *M*_6_ respectively denotes left SCM, left UT, left CE, right CE, right UT and right SCM. The *A*_1_, *A*_2_, *A*_3_, *A*_4_, *A*_5_, *A*_6_ and *A*_7_ respectively denotes low head, head backwards, left flexion, right flexion, left rotation and right rotation, hands up. (**a**) The number distribution of features in muscles and movements. A square represent the number of feature on *M*_*i*_ activated by *A*_*i*_. The darker the square’s color is, the more the features on the square are. (**b**) The importance distribution of features in muscles and movements. A square represent the importance of feature on *M*_*i*_ activated by *A*_*i*_. The darker the square’s color is, the more important the features on the square are.

In order to further analyze the feature distribution above, the number distribution and importance distribution of the features in the muscles and movements are plotted as units of the heat map in Fig. 4. The darker the color of the unit is in Fig. 4, the more the features on the unit are in Fig. 4(a), and the more important the features on the unit are in Fig. 4(b). As shown in Fig. 4, the features on the units formed by the *M*_3_ (left CE) and *A*_1_ (bow), *M*_4_ (right CE) and *A*_1_ (bow), *M*_2_ (left UT) and *A*_7_ (hands up), *M*_5_ (right UT) and *A*_7_ (hands up) are the most in number and most important for the CS identification performance. It is concluded that the CE activated by the movement of *A*_1_ (bow head) and the UT activated by the movement of *A*_7_ (hands up) may be more closely related to the CS.

**Figure 5:**
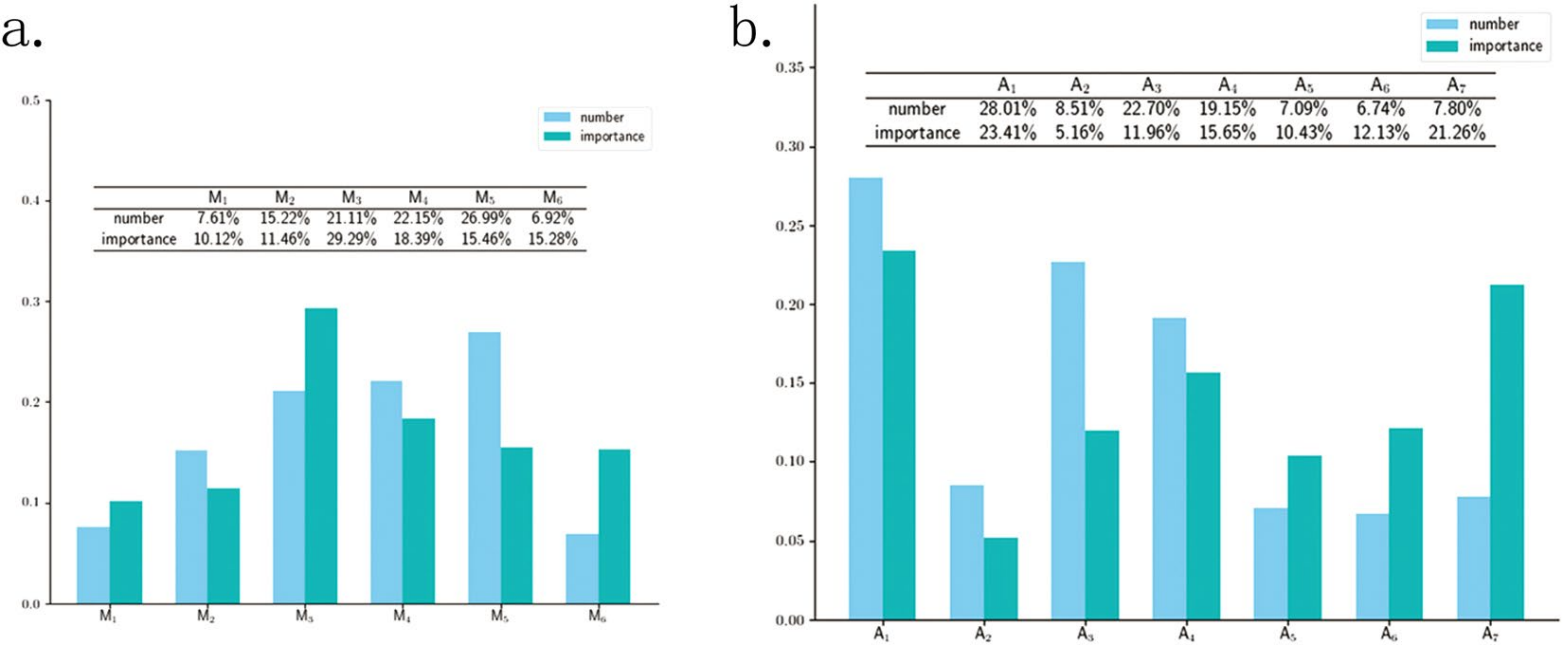
The feature distribution. (**a**) The feature distribution on the muscles. The *M*_1_, *M*_2_, *M*_3_, *M*_4_, *M*_5_, *M*_6_ respectively denotes left SCM, left UT, left CE, right CE, right UT and right SCM. The light bars show the distribution of feature numbers (A-distribution). The right UT rank first, followed by right CE, left CE, left UT, left SCM, and right SCM. The right UT, right CE and left CE take up 70.25%. The dark bars show the distribution of feature importance (B-distribution). The left CE rank first, followed by right CE, right UT, right SCM, left UT and left SCM. The left CE, right CE and right UT take up 63.14%. (**b**) The feature distribution on the movements. The *A*_1_, *A*_2_, *A*_3_, *A*_4_, *A*_5_, *A*_6_ and *A*_7_ respectively denotes low head, head backwards, left flexion, right flexion, left rotation, right rotation and hands up. The light bars show the distribution of numbers (C-distribution). The movement of head backwards rank first, followed by left flexion, right flexion, low head, hand up, left rotation, right rotation. The dark bars show the distribution of feature importance (D-distribution). The movement of the bow head rank first, followed by hands up, right flexion, right rotation, left flexion, left rotation, head backwards.

In order to further understand feature distribution, the feature number distribution in muscles (A-distribution), the importance distribution in muscles (B-distribution), the feature number distribution in movements (C-distribution), and the features importance distribution in movements (D-distribution), are plotted as Fig. 5. As shown in Fig. 5(a), the features on UT (*M*_2_ and *M*_5_) and CE (*M*_3_ and *M*_4_) take up 85.47% in the A-distribution and 74.60% in the B-distribution. As shown in Fig. 5(b), the features on the *A*_1_ (bow) rank first in both C-distributions and D-distribution. The features on *A*_7_ (hands up) rank second in D-distribution. It is concluded that the CE play an most important role in identifying CS followed by UT. And the movements of *A*_1_ (bow head) also play an most importance role in identifying CS, followed by the movement of *A*_7_ (hands up).

The conclusion can be explained by that the more frequently muscle are activated, the more the muscle (movement) is related to CS. Most of volunteers are sedentary population who frequently bow head and use the hands and arms to work. The movement of bowing head activate the CE, and the movement of the hands and arms activate the UT. The excessive use of the CE and UT cause muscle strain, cause dysfunction of the spine stabilizing system, and accelerate cervical degeneration and lead to CS. It may provide a suggestion for sedentary population to prevent CS by avoiding excessive use of the CE and UT, strengthen the UT and CE protection.

## Discussion

The CS identification methods mainly include clinical symptoms examination and imaging examination since the diagnosis of CS is determined by clinical symptoms and imaging information^16,17^. Currently, the clinical symptoms examination are performed by experts or doctors in the form of the inquiry. The images examination, which mostly includes spinal angiography, vertebral artery angiography, X-ray, computed to-mography (CT), and magnetic resonance imaging (MRI), etc, mainly depend on observation of the physical changes in spine and its subsidiary structure by imaging instruments to identify CS. The EasiCNCSII depend on detecting abnormal sEMG signal associated with muscle activity to identify CS. We compare the practicality of the EasiCNCSII with the inquiry, imaging method as shown in Table 3. The inquiry method is easiest and fastest. However, the method is suitable for population with obvious symptoms, for example severe pain, since information is mainly determined by suffer’s subjective feelings and judgment. And it needs the instruction of the doctor and auxiliary equipment to accurately identify CS. The images examination is the essential CS examination currently. However, its cost is relatively high and it is time-consuming considering time spent to go to the hospital and wait for the results which requires the intervention of doctors or experts. What’s more, it can not be used frequently with concern on health since the frequent use of imaging instruments can put a strain on the health, for example the radiation. Compared with inquiry and imagines examination, the EasiCNCSII is an best choice to identify CS outside the clinic with the advantage of easy use, low cost, no-harm. Owing to the intelligent algorithm, users can get results quickly after examination. What’s more, the wearable sEMG acquisition technology get more and more attention^65^. Combined with mobile application, the EasiCNCSII potentially provide low-cost convenient universal access to indispensable care outside the clinic, and even promote the development of telemedicine, especially in areas short of medical resources.

**Table 3:** The comparison of the Inquiry, Images examination, EasiCNCSII.

	Inquiry	Images examination	EasiCNCSII
Time	dozens of minutes	dozens of minutes	about 20 minutes
Cost	low	high	middle
Harmful	no	yes	no
Location	hospital	hospital	no limite
Convenience	low	low	high
Doctor/professional	yes	yes	no
The time of getting the results	a few minutes	days	several seconds

To our best knowledge, previous research on CS identification based on the sEMG and machine learning are few, so there is a lot room for improvement. Traditional classification or regression algorithms can achieve good performance when fed with a wealthy of high quality data. However, the amount of data is limited and the data dimension is high. Besides, data acquisition is vulnerable to the environment so that poor quality data are often collected by portal sEMG. The EasiAI can handle these influences by boosting multiple weak learners to reach higher prediction accuracies. Compared with the RF, SVM, LR and NB, the EasiAI achieves best performance, and identifies complex crowd with low missed diagnosis rate. It is hard to fully understand the relation between CS and the sEMG signal from the activity of the deep and shallow muscles with the limited data, but the data-driven machining learning can achieve better performance and accelerate our understanding of the principles behind the CS identification to assist diagnosis and guide treatment as more data are accumulated. We are looking forward to more convenient and intelligent applications in CS studies.

## Conclusion

We have proposed the convenient non-harm CS intelligent identify method EasiCNCSII which includes data acquisition method and intelligent CS identification algorithms. It is able to collect data in a harmless and convenient method in a short period of time and identity the CS with an encouraging performance. To achieve the best performance on the limited data set of weak availability, we developed a data processing framework consisting of feature extraction, feature selection after the extraction, and the intelligent recognition algorithm. Combined with mobile application, the EasiCNCSII potentially provide low-cost convenient universal access to indispensable care outside the hospital, especially in the remote rural areas with poor medical resources, which potentially promotes a balanced distribution of quality medical resources. Our future work will focus on collecting and building the more larger database including sEMG signal data and other CS-related information, and implement an overall high-performance CS identification application system.

## Electronic supplementary material

Supplementary Information

## Data Availability

The raw sEMG data supporting this study are not publicly available due to user privacy, but are available from the corresponding author on reasonable request. However, the data set, which are extracted from raw sEMG data by the feature extraction methods in this paper, support automatic CS identification study, are available at github after reorganized, each sample of which consist of 2949 features. Currently, it is available from wangnana@ict.ac.cn on reasonable request.
