# Supplementary material for: A Convenient Non-harm Cervical Spondylosis Intelligent Identity method based on Machine Learning
Source: Sci Rep. 2018 Nov 27;8:17430. doi: 10.1038/s41598-018-32377-3 (PMC6258664; doi:10.1038/s41598-018-32377-3)
Supplement: Supplementary file 1 — Supplementary Information [file 41598_2018_32377_MOESM1_ESM.pdf]

SUPPLEMENTARY INFORMATION
A Convenient Non-harm Cervical Spondylosis Intelligent Identity method based on Machine Learning

Nana Wang, Xi Huang, Yi Rao, Jing Xiao, Jiahui Lu, Nian Wang, Li Cui

Supplementary Methods

The selection of muscles and movements In order to identify CS, the selected muscles should meet the following requirements. Firstly, the selected muscles are associated with the CS. It is known that CS belongs to tendons injury in traditional Chinese medicine. It is supported by the tendons injury theory in traditional Chinese medicine that cervical soft tissue abnormalities cause CS, and the tender points are the indication of cervical soft tissue abnormalities[1,2]. The tender points of CS mostly focus on the cervical paravertebral muscle, trapezius and sternocleidomastoid muscle[3]. Thus, the cervical paravertebral muscle, trapezius and sternocleidomastoid muscle are competitive choice. Secondly, the selected muscles can be activated by the activities, producing sEMG signal. The cervical erector spinae (CE) and the sternocleidomastoid (SCM) participate in most of the neck basic function activities above. The trapezius is activated by the scapula activities which mainly includes shoulder and hand movements, especially the upper trapezius (UT). Thus, the CE, SCM and UT are suitable choice. Finally, the sEMG signal generated by the selected muscles can be easily acquired with the minimal interference. The guidelines for electrode placement[4–7] provide the mature surface electrodes location for CE, UT and SCM. Thus, we select the left sternocleidomastoid(M_1), left upper trapezius(M_2), left cervical erector spinae (M_3), right cervical erector spinae(M_4), the right upper trapezius(M_5), the right sternocleidomastoid(M_6) (see Supplementary Figure S_1 for the location of surface electrodes) as collected muscles.

In order to identify CS, the selected movements should meet the following requirement. The movement can maximally activate muscles to produce the most obvious sEMG signal which is easily collected by sEMG device and have the most significant difference between CS suffer and the healthy free from the CS. Since it is hardly to fully understand the principles between sEMG signals and the activity of the deep and shallow muscles to our best knowledge, so all the movement activating the muscles above need to be considered. The function activities, which CE, SCM and UT are involved in, are mainly rotation, lateral flexion, bow, head backwards and scapula activities.

So we select 7 representative movements from the functional activities above which consist of the following movements: bow(A_1), head backwards(A_2), left flexion(A_3), right flexion(A_4), left rotation(A_5), right rotation(A_6), hands up(A_7).

The instruction of data acquisition We use the 6-channel sEMG device as shown in Figure S_2, each channel of which is connected to fixed muscle. The 6-channel sEMG device can simultaneously collect 6 sEMG signals from 6 muscles with a sampling frequency of up to 1062 Hz.

We acquire the data according to the following instruction. Firstly, we connect the sEMG device to laptop. Secondly, the selected muscles are connected with sEMG device by surface electrodes according to the Figure S_1, following careful skin preparation. Then, the subject perform the movements in the order of bow(A_1), head backwards(A_2), left flexion(A_3), right flexion(A_4), left rotation(A_5), right rotation(A_6), hands up(A_7) according to the following instruction. The detail instruction is as follow:

- Standing: open your feet so that the distance between your feet is shoulder width. Keep head up, eyes looking straight ahead, and arms naturally hanging by sides.
- A_1: stand for 5 seconds, bow to the maximum extent for 5 seconds, hold for 5 seconds, revert head to neutral position.
- A_2: stand for 5 seconds, bend the head backwards to the maximum extent for 5 seconds, hold for 5 seconds, revert head to neutral position.
- A_3: stand for 5 seconds, flexion to the left to the maximum extent for 5 seconds, hold for 5 seconds, revert head to neutral position.
- A_4: stand for 5 seconds, flexion to the right to the maximum extent for 5 seconds, hold for 5 seconds, revert head to neutral position.
- A_5: stand for 5 seconds, rotate to the left to the maximum extent for 5 seconds, hold for 5 seconds, revert head to neutral position.
- A_6: stand for 5 seconds, rotate to the right to the maximum extent for 5 seconds, hold for 5 seconds, revert head to neutral position.

- A_7: stand for 5 seconds, raise hands to the maximum extent for 5 seconds, hold for 5 seconds, put hands on both sides of the body. Each movement is held for 20 seconds and repeated 3 times with 5 seconds rest between each repetition. In the data acquisition process, subject keeps still below shoulder. Finally, the sEMG activity from the upper trapezius (UT), cervical erector spinae (CE) and the sternocleidomastoid (SCM) are recorded bilaterally with Ag/AgCl surface electrodes. And the sEMG signal is converted to digital signal and sent to the laptop by the sEMG device.

Feature extraction The 5 kinds of common feature extraction methods, which include time-domain, frequency-domain, time-frequency, parametric model and nonlinear feature analysis, are used to extract features from the sEMG signal. The methods of using the disease-related knowledge to build features are also considered. We respectively extract 63 common features from the $S_{i,j}$ $(1 \leq i \leq 7, 1 \leq j \leq 6)$ in the 5 kinds of common feature extraction methods above. And we respectively extract 43 from the S_i inspired by relevant research and pathology. Besides, the FRR features are also extracted from S_1. Thus, 2949($(63 \times 6 + 45) \times 1 + (63 \times 6 + 43) \times 6)$ features are extracted from the raw sEMG signal of a subject. For the convenience of statistical analysis, we divided the 108 kinds of features above into 11 types of combination features, which are TF, FF, WL, WLP, AR, EN, FRR, DU, ACI, UN, SYM as shown in table S_1.

We introduce feature extraction process in detail as shown in table S_2. The RMS, AEMG, IEMG, MF and MPF are computed by formula 1-7. The minimum and maximum values, the mean value, variance, skewness, kurtosis, autocorrelation sequence and the peaks of the Discrete Fourier transform (DFT) of $S_{i,j}$ with the corresponding frequencies are extracted in methods of time-domain and frequency-domain[8,9]. The 23 features are extracted from $S_{i,j}$ in time-frequency methods including the methods of wavelet transform and wavelet-package transform, of which 15 are computed in methods of wavelet transform[10] and 8 are computed in methods of wavelet-package transform[11]. Meanwhile, Meanwhile, 14 features from $S_{i,j}$ are computed in parametric model methods AR[12], as well as 1 features in nonlinear methods of entropy analysis[13].

The FRR, DU, ACI, UN, SYM are extracted. The flexion relaxation ratio (FRR) is a useful, reliable marker to show altered neuromuscular function in both chronic neck pain patients and controls[14,15]and can only be extracted from the S_1 (the data from A_1). We refer to the calculation method of the FRR in the paper[16] and extracted the FRR from S_1. To our best knowledge, the DU,

ACI, UN, SYM are firstly proposed to identify CS in our paper. According to formula 8, the DU is computed as the duration of muscle activation inspired by that the flexion relaxation phenomenon (FRP) which refers to a reduced or sudden onset of myoelectric silence in erector spinae muscles during full trunk flexion[17]. As shown in formula 9-12, we build new feature ACI based on that population with neck pain have an altered pattern of muscle activation in the deep and superficial cervical flexor muscles[18,19]. Besides, inspired by the imbalanced index proposed by Oddsson[20] and cross syndrome, the UN that include RMS ratio between different muscles was put forward in our work as shown in formula 13-22[20]. In order to explore the characteristics of bilateral muscle movement patterns, The SYM are extracted by calculating the similarity of the raw sEMG signal between symmetrical muscles using European distance, shape-based distance(SBD)[21], dynamic time warping(DTW)[22] according to formula 23-25.

1. Zhongmin, Z. *Study on the Symptoms and Soft Tissue Changes of Cervical spondylosis*. Ph.D. thesis, China Academy of Chinese Medical Sciences (2011).

2. Xiaoqian, Y. *Clinical observation of common tender point in cervical spondylosis*. Master's thesis, Beijing University of Chinese Medicine (2010).

3. association of rehabilitation medicine, C. Guide to diagnosis and treatment of cervical spondylosis. Tech. Rep., Chinese association of rehabilitation medicine (2010).

4. CM, S., SM, J., V, H. & SD, M. Use of surface electromyography to estimate neck muscle activity. *Journal of electromyography and kinesiology:official journal of the International Society of Electrophysiological Kinesiology* **10**, 377 (2000).

5. SENIAM. Recommendations for sensor locations on individual muscles. `http://www.seniam.org/`. Accessed February 12, 2018.

6. Hermens, H. J. *et al.* European recommendations for surface electromyography. *Roessingh research and development* **8**, 13–54 (1999).

7. Falla, D., Dall'Alba, P., Rainoldi, A., Merletti, R. & Jull, G. Location of innervation zones of sternocleidomastoid and scalene muscles–a basis for clinical and research electromyography applications. *Clinical Neurophysiology* **113**, 57–63 (2002).

8. Altun, K., Barshan, B. & Tunçel, O. Comparative study on classifying human activities with miniature inertial and magnetic sensors. *Pattern Recognition* **43**, 3605–3620 (2010).

9. Yüksek, M. C. *A comparative study on human activity classification with miniature inertial and magnetic sensors*. Ph.D. thesis, bilkent university (2011).

10. Chowdhury, S. K., Nimbarte, A. D., Jaridi, M. & Creese, R. C. Discrete wavelet transform analysis of surface electromyography for the fatigue assessment of neck and shoulder muscles. *Journal of Electromyography and Kinesiology* **23**, 995–1003 (2013).

11. Babita, Kumari, P., Narayan, Y. & Mathew, L. Binary movement classification of semg signal using linear svm and wavelet packet transform. In *IEEE International Conference on Power Electronics, Intelligent Control and Energy Systems*, 1–4 (2017).

12. Cui, J. G., Wang, X., Zhong hai, L. I. & Tian, F. The method of surface emg pattern recognition based on ar parameter model and clustering analysis. *Acta Metrologica Sinica* **27**, 286–289 (2006).

13. Chen, W. T., Wang, Z. Z., Xiao, H. U. & Xiao-Pu, L. I. Entropy analysis of semg signal during dynamic contractions for assessing muscle fatigue. *Chinese Journal of Medical Physics* (2006).

14. Murphy, B. A., Marshall, P. W. & Taylor, H. H. The cervical flexion-relaxation ratio: reproducibility and comparison between chronic neck pain patients and controls. *Spine* **35**, 2103–2108 (2010).

15. Shin, S.-j., An, D.-h., Oh, J.-s. & Yoo, W.-g. Changes in pressure pain in the upper trapezius muscle, cervical range of motion, and the cervical flexion–relaxation ratio after overhead work. *Industrial health* **50**, 509–515 (2012).

16. Murphy, B. A., Marshall, P. W. & Taylor, H. H. The cervical flexion-relaxation ratio: reproducibility and comparison between chronic neck pain patients and controls. *Spine* **35**, 2103–2108 (2010).

17. Floyd, W. & Silver, P. The function of the erectores spinae muscles in certain movements and postures in man. *The Journal of physiology* **129**, 184–203 (1955).

18. Falla, D. Unravelling the complexity of muscle impairment in chronic neck pain. *Manual therapy* **9**, 125–133 (2004).

19. Falla, D., Bilenkij, G. & Jull, G. Patients with chronic neck pain demonstrate altered patterns of muscle activation during performance of a functional upper limb task. *Spine* **29**, 1436–1440 (2004).

20. Oddsson, L. I., Giphart, J. E., Buijs, R. J., Roy, S. H. *et al.* Development of new protocols and analysis procedures for the assessment of LBP by surface EMG techniques. *Journal of rehabilitation research and development* **34**, 415 (1997).

21. Paparrizos, J. & Gravano, L. k-shape: Efficient and accurate clustering of time series. In *Proceedings of the 2015 ACM SIGMOD International Conference on Management of Data*, 1855–1870 (ACM, 2015).

22. Yamauchi, T., Xiao, K., Bowman, C. & Mueen, A. Dynamic time warping: A single dry electrode eeg study in a self-paced learning task. In *Affective Computing and Intelligent Interaction (ACII), 2015 International Conference on*, 56–62 (IEEE, 2015).

23. Duda, R. O., Hart, P. E. & Stork, D. G. *Pattern classification* (John Wiley & Sons, 2012).

24. Nie, F., Xiang, S., Jia, Y., Zhang, C. & Yan, S. Trace ratio criterion for feature selection. In *AAAI*, vol. 2, 671–676 (2008).

25. Brown, G., Pocock, A., Zhao, M.-J. & Luján, M. Conditional likelihood maximisation: a unifying framework for information theoretic feature selection. *Journal of machine learning research* **13**, 27–66 (2012).

26. Nie, F., Huang, H., Cai, X. & Ding, C. H. Efficient and robust feature selection via joint l2, 1-norms minimization. In *Advances in neural information processing systems*, 1813–1821 (2010).

27. Cai, D., Zhang, C. & He, X. Unsupervised feature selection for multi-cluster data. In *Proceedings of the 16th ACM SIGKDD international conference on Knowledge discovery and data mining*, 333–342 (ACM, 2010).

28. Yang, Y., Shen, H. T., Ma, Z., Huang, Z. & Zhou, X. l2, 1-norm regularized discriminative feature selection for unsupervised learning. In *IJCAI proceedings-international joint conference on artificial intelligence*, vol. 22, 1589 (2011).

29. Li, Z. *et al.* Unsupervised feature selection using nonnegative spectral analysis. In *AAAI*, vol. 2, 1026–1032 (2012).

30. Li, J. *et al.* Feature selection: A data perspective. *ACM Computing Surveys (CSUR)* **50**, 94 (2017).

31. xgboost developers. Xgboost parameters. `http://xgboost.readthedocs.io/en/latest/parameter.html`. Accessed July 20, 2018.

32. scikit-learn developers. Sklearn ensemble svc. `http://scikit-learn.org/stable/modules/generated/sklearn.svm.SVC.html`. Accessed July 20, 2018.

33. scikit-learn developers. Sklearn ensemble logisticregression. `http://scikit-learn.org/stable/modules/generated/sklearn.linear_model.LogisticRegression.html`. Accessed July 20, 2018.

34. scikit-learn developers. Sklearn ensemble gaussiannb. `http://scikit-learn.org/stable/modules/generated/sklearn.naive_bayes.GaussianNB.html`. Accessed July 20, 2018.

35. scikit-learn developers. Sklearn ensemble randomforestclassfier. `http://scikit-learn.org/stable/modules/generated/sklearn.ensemble.RandomForestClassifier.html`. Accessed July 20, 2018.

Formula

$$mean_i = \frac{1}{n} \times \sum_{j=1}^{n} p_{i,j} \tag{1}$$

$$RMS = \begin{bmatrix} rms_1, & rms_2, & rms_3, & rms_4, & rms_5, & rms_6 \end{bmatrix} \tag{2}$$

$$rms_i = \sqrt{\sum_{j=1}^{n} (p_{i,j} - mean_i)^2} \tag{3}$$

$$AEMG = \begin{bmatrix} aemg_1, & aemg_2, & aemg_3, & aemg_4, & aemg_5, & aemg_6 \end{bmatrix} \tag{4}$$

$$aemg_i = \frac{1}{n} \times \sum_{j=1}^{n} |p_{i,j} - mean_i| \tag{5}$$

$$IEMG = \begin{bmatrix} iemg_1, & iemg_2, & iemg_3, & iemg_4, & iemg_5, & iemg_6 \end{bmatrix} \tag{6}$$

$$iemg_i = \sum_{j=1}^{n} |p_{i,j} - mean_i| \tag{7}$$

$$DU = |N_i| \tag{8}$$

$$ACI = \begin{bmatrix} AC_1, & AC_2, & AC_3, & AC_4, & AC_5, & AC_6, & COM \end{bmatrix} \tag{9}$$

$$AC_i = \frac{COM_i}{COM} \tag{10}$$

$$COM_i = \sum_{j=1}^{n} |p_{i,j}| \tag{11}$$

$$COM = \sum_{i=1}^{6} \sum_{j=1}^{n} |p_{i,j}| \tag{12}$$

$$UN = \begin{bmatrix} UNB, & UT_s, & SCM_s, & SU, & SC, & CU, & RS \end{bmatrix} \tag{13}$$

$$UNB = \begin{bmatrix} unbalance(1,2), & unbalance(1,3) & ,..., & unbalance(1,6) \end{bmatrix} \tag{14}$$

$$unbalance_{i,j} = \frac{rms_i}{rms_j} \tag{15}$$

$$UT_s = rms_1 + rms_4 \tag{16}$$

$$SCM_s = rms_0 + rms_5 \tag{17}$$

$$CE_s = rms_2 + rms_3 \tag{18}$$

$$SU = \frac{|UT_s - SCM_s|}{|UT_s + SCM_s|} \tag{19}$$

$$SC = \frac{|SCM_s - CE_s|}{|SCM_s + CE_s|} \tag{20}$$

$$CU = \frac{|CE_s - UT_s|}{|CE_s + UT_s|} \tag{21}$$

$$RS = \begin{bmatrix} (rms_0 - rms_5), & (rms_1 - rms_4), & (rms_2 - rms_3) \end{bmatrix} \tag{22}$$

$$SYM = \begin{bmatrix} ED, & SDB, & DTW, & Max(ED), & Max(SDB), & Max(DTW) \end{bmatrix} \tag{23}$$

$$ED = \begin{bmatrix} ED(N_1, N_6), & ED(N_2, N_5), & ED(N_3, N_4) \end{bmatrix} \tag{24}$$

$$ED(i, j) = \sqrt{\sum_{i=1}^{n} (p_{k,i} - p_{6-k,i})^2} \tag{25}$$

Table S1: Feature type

	TF	FF	WL	WLP	AR	EN	FRR	DU	ACI	UN	SYM
Dimension	11	14	15	8	14	1	2	1	7	23	12

Table S2: The features and related information

	Name	Dimension	Calculation
	RMS	1	formula 1-3
	AEMG	1	formula 4-5
	IEMG	1	formula 6-7
	MEAN	1	
	VAR	1	
TF	STD	1	
(time-domain)	Mode	1	
	MAX	1	
	MIn	1	
	over-zero	1	
	Time-range	1	
	DC	1	
	SHAPE-MEAN	1	
	SHAPE-STD-2	1	
	SHAPE-STD	1	
	SHAPE-SKEW	1	
	SHAPE-KURT	1	
FF	fft-MEAN	1	frequency-domain
(frequency-domain)	fft-var	1	
	fft-std	1	
	fft-skew	1	
	fft-kurt	1	
	fft-entropy	1	
	MF	1	
	MPF	1	

WL(time-frequency)	wavelet	15	
WLP(time-frequency)	wavelet-packet	8	
AR(parameter model)	ar	14	
EN(nonlinear analysis)	entropy	1	
FRR	frr	2	
DU	duration	1	formula 8
ACI	ACI	7	formula 9-12
UN	UN	23	formula 13-22
SYM	SYM	12	formula 23-25

Table S3: The comparison of performance with different feature selection algorithms.

	Accuracy	Sensitivity	Specificity	FNR	FPR	Feature Number	Type
Non	86.54%	92.51%	77.14%	7.49%	22.86%	2949	
FS	87.10%	95.28%	74.29%	4.72%	25.71%	282	supervised
TRC	87.10%	95.28%	74.29%	4.72%	25.71%	282	supervised
CIFE	86.00%	92.60%	75.71%	7.40%	24.29%	282	supervised
DISR	82.06%	91.65%	67.14%	8.35%	32.86%	282	supervised
REFS	84.89%	91.69%	74.29%	8.31%	25.71%	282	supervised
MCFS	83.16%	92.60%	68.57%	7.45%	31.43%	282	unsupervised
UDFS	74.81%	84.29%	60.00%	15.71%	40.00%	282	unsupervised
NDFS	76.52%	84.42%	64.29%	15.58%	35.71%	282	unsupervised
F-score	87.10%	95.28%	74.29%	4.72%	25.71%	282	supervised
GI	48.03%	77.01%	2.86%	22.99%	97.14%	282	supervised
CFS	64.16%	72.25%	51.43%	27.75%	48.57%	5	supervised
easiRF	91.02%	97.14%	81.43%	2.86%	18.57%	282	supervised

1 Similarity based Methods: Fisher Score(FS, in short)[23], Trace Ratio Criterion(TRC, in short)[24].

2 Information Theoretical based Methods: Conditional Infomax Feature Extraction(CIFE)[25], Double Input Symmetrical Relevance(DISR)[25].

3 Sparse Learning based Methods: Efficient and Robust Feature Selection (REFS)[26], Multi-Cluster Feature Selection (MCFS)[27], l2, 1-Norm Regularized Discriminative Feature Selection(udfs)[28], Non-negative Discriminative Feature Selection(NDFS)[29].

4 Statistical based Methods[30]: F-score, Gini Index(GI), CFS.

5 Without affecting the performance of the algorithms, the number of features selected by the algorithms above except CFS, the features number of which automatically generated, is respectively set as 282.

Table S4: The parameters of model EasiAI.

Parameters \ Folds	1_th	2_th	3_th	4_th	5_th
$booster$	$gbtree$				
$objective$	$binary : logistic$				
$eval\_metric$	auc				
$gamma$	0	0	10	0	0
max_depth	6	5	9	5	8
$alpha$	0	0	0	0	1
sub_sample	0.8	0.7	0.8	0.7	0.49
$colsample\_bytree$	0.7	0.8	0.8	0.8	0.8
$colsample\_bylevel$	0.8				
eta	0.01	0.01	0.01	0.01	0.12
$tree\_method$	exact				
$seed$	0				

* $booster$: base classifier. $eval\_metric$: the metric is used for validation data. max_depth: the maximum depth of a tree. sub_sample: the percentage of the total number of samples. $colsample\_bytree$: the percentage of the total number of features. eta: learning rate. num_boost_round: the number of trees. See reference[31] for details on parameters of GDBT.

Table S5: The parameters of model SVM.

Parameters \ Folds	1_th	2_th	3_th	4_th	5_th
c	0.2	1	0.2	0.90	0.15
$gamma$	0.001	0.006	0.001	0.006	0.001
$kernel$	rbf				
$degree$	3				
$coef0$	0.0				
$probability$	False				
$shrinking$	True				
tol	1e-3				

* See reference[32] for details on parameters of the support vector machines(SVM).

Table S6: The parameters of model LR.

Folds / Parameters	1_th	2_th	3_th	4_th	5_th
$solver$	soga				
$penalty$	l2				
$fit\_intercept$	True				
C	1.0				
$random\_state$	10				

* See reference[33] for details on parameters of the Logistic regression(LR).

Table S7: The parameters of model NB.

Folds / Parameters	1_th	2_th	3_th	4_th	5_th
$priors$	None				

* See reference[34] for details on parameters of the Native-Bayes(NB).

Table S8: The parameters of model RF.

Folds / Parameters	1_th	2_th	3_th	4_th	5_th
$criterion$	entropy	gini	gini	entropy	gini
$max\_features$	auto				
max_depth	none				
$n\_estimators$	230	220	410	90	350
oob_score	True				

* See reference[35] for details on parameters of the Random Forest(RF).

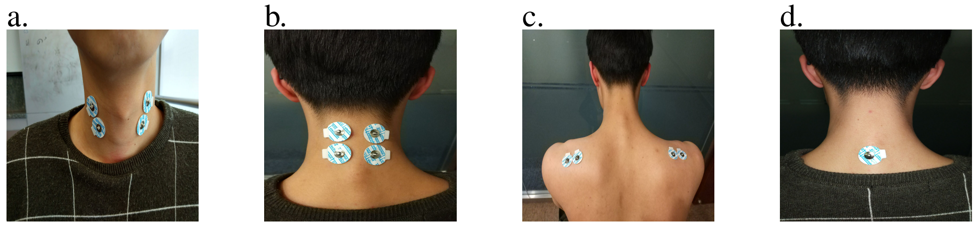

Figure S1: The Location of surface electrodes. (a), the sternocleidomastoid (SCM): the one third of the line from sternal to mastoid. (b), the cervical erector spinae (CE): the 2cm from the spine of c3-c6. (c), the upper trapezius (UT): The 50% on the line from the acromion to the spine on vertebra C7. (d), reference electrode: on vertebra C7.

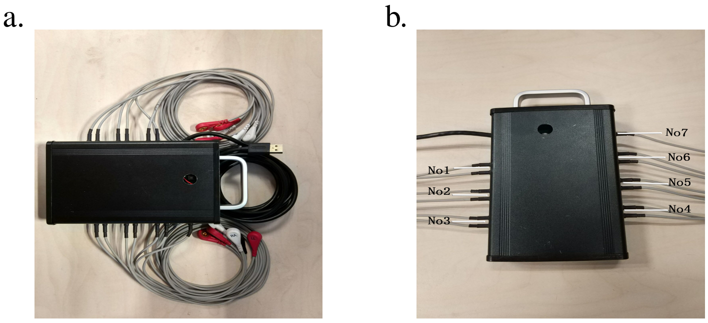

Figure S2: The sEMG device. No1 is connected to the left sternocleidomastoid. No2 is connected to the left upper trapezius. No3 is connected to the left cervical erector spinae. No4 is connected to the right cervical erector spinae. No5 is connected to the right upper trapezius. No6 is connected to the right sternocleidomastoid. No7 is connected to spin of C7 spinous.

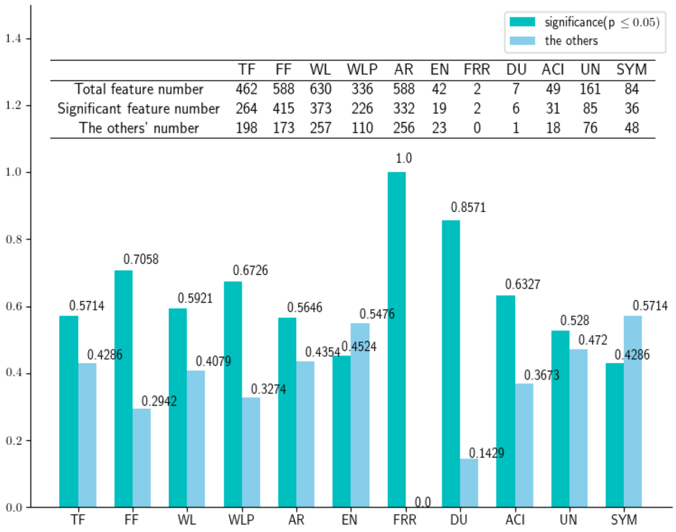

	TF	FF	WL	WLP	AR	EN	FRR	DU	ACI	UN	SYM
Total feature number	462	588	630	336	588	42	2	7	49	161	84
Significant feature number	264	415	373	226	332	19	2	6	31	85	36
The others' number	198	173	257	110	256	23	0	1	18	76	48

Figure S3: The 2949 features of samples associated with the CS.

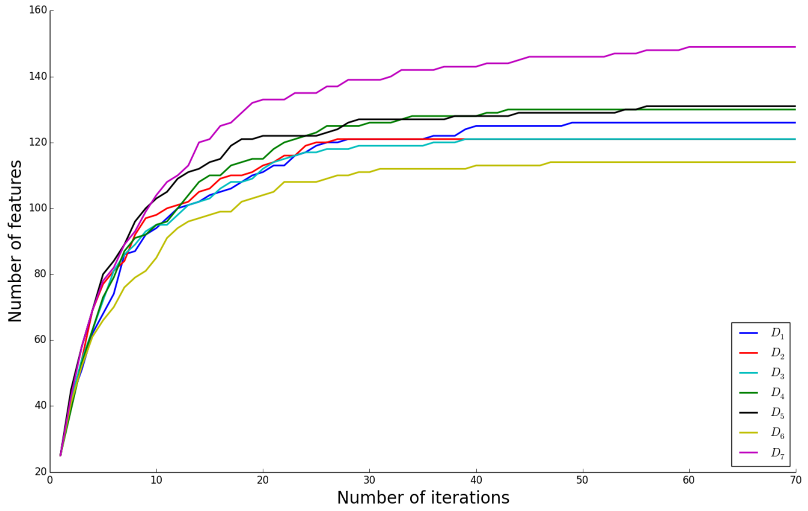

Figure S4: The feature selection. Different colors indicate different data sets. The x-axis represents the number of iterations. The y-axis represents the number of selected features. When the number of iterations is less than 40 the number of features increases exponentially. When the number of iterations is greater than 40, the number of features increases slowly and tends to be stable.

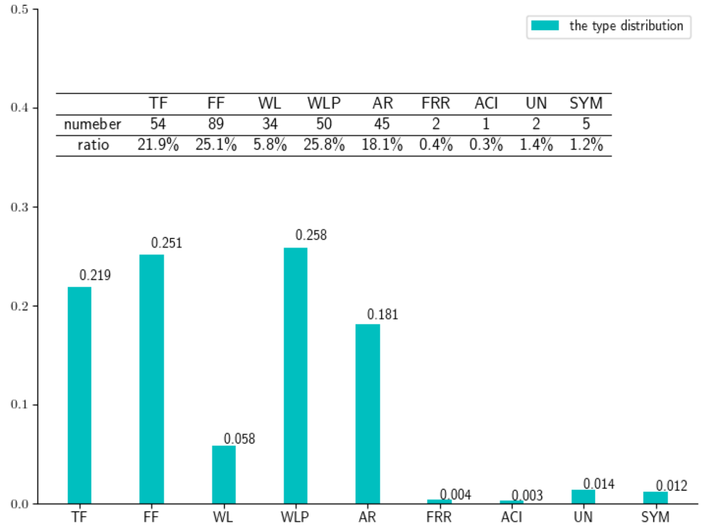

Figure S5: The feature type distribution of 282 features. The 282 features are distributed on 11 types: TF, FF, WL, WLP, AR, EN, FRR, DU, ACI, UN, SYM.

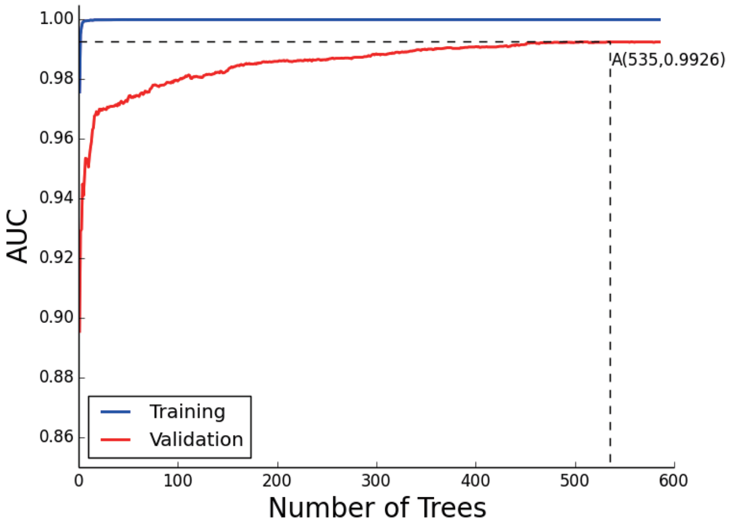

Figure S6: Effect that the number of regression trees included in EasiAI (x-axis) has in the prediction AUC (y-axis). The red curve denotes the effect that trees number has in the prediction AUC on validation set. The blue curve denotes the effect that trees number has in the prediction AUC on training set.
